# Subunit C of V-ATPase-VmaC Is Required for Hyphal Growth and Conidiation in *A. fumigatus* by Affecting Vacuolar Calcium Homeostasis and Cell Wall Integration

**DOI:** 10.3390/jof8111219

**Published:** 2022-11-17

**Authors:** Congcong Sun, Xingyue Li, Yuanwei Zhang, Ling Lu

**Affiliations:** Jiangsu Key Laboratory for Microbes and Functional Genomics, College of Life Sciences, Nanjing Normal University, Nanjing 210023, China

**Keywords:** Vacuolar H^+^-ATPase (V-ATPase), calcium homeostasis, cell wall integration, *Aspergillus fumigatus*

## Abstract

*Aspergillus fumigatus* is a widespread airborne fungal pathogen in humans. However, the functional genes in *A. fumigatus* that may contribute to its pathogenesis have not yet been fully identified. Vacuolar H^+^-ATPase is universal in eukaryotic organisms but exhibits specific roles in various species. Here, we identified VmaC as a putative subunit of vacuolar H^+^-ATPase in *A. fumigatus* that is widely conserved through evolution. The C-terminal hydrophobic domain of VmaC plays a critical role in its vacuolar localization and growth and conidiation. Deletion or turn-off of VmaC encoding gene-*AfvmaC* expression is not lethal but leads to a very sick and tiny colony phenotype, which is different from that of yeast with conditional *ScvmaC* defects. Furthermore, we found that *AfvmaC* not only participates in maintaining calcium homeostasis and vacuolar acidity but is also involved in cell wall integration pathway regulation, highlighting the importance of the vacuole as a storage organelle associated with many aspects of cellular homeostasis. This study indicates that fungal VmaC is relatively conserved. When compared to that in model yeasts, VmaC in *A. fumigatus* is required for hyphal growth and conidiation, suggesting that specific motifs in VmaC might be functioned in *Aspergilli*.

## 1. Introduction

Invasive aspergillosis (IA) has emerged as one of the most common life-threatening fungal diseases affecting immunocompromised individuals, with mortality rates as high as 90% [1]. IA is predominantly caused by *Aspergillus fumigatus*, a worldwide environmental and airborne fungal pathogen in humans [2]. *Aspergillus* infection is on the rise due to the use of antibiotics and immunosuppressants, causing great concern in the medical community [3]. Currently available antifungal drugs to treat IA are limited because of increasing drug resistance and side effects/toxicities [4]. Recent developments in *A. fumigatus* have heightened the need for examining functional genes that contribute to its pathogenesis and may provide novel drug targets.

Fungal vacuoles are acidic compartments that play numerous roles, including amino acid storage and phosphate, pH and ion homeostasis maintenance [5,6]. Vacuole-type H^+^-ATPase (V-ATPase) is found in almost every eukaryotic cell and is responsible for supplying energy to various internal organelles and membrane assemblies [7]. V-ATPase produces proton motive force by hydrolyzing ATP. The energy provided by V-ATPase promotes the acidification of intracellular compartments in eukaryotic cells and plays a crucial role in receptor-mediated endocytosis, protein degradation and intracellular trafficking processes [8,9]. V-ATPase is a multisubunit protein consisting of two distinct functional domains, V_1_ and V_0_. The V_1_ domain consists of eight subunits, designated A–H, which are encoded by the yeast *vma1*, *vma2*, *vma5*, *vma8*, *vma4*, *vma7*, *vma10* and *vma13* genes, respectively. The V_0_ domain is composed of six different subunits (a, d, e, c, c′, c′′), which are encoded by the *vph1* (or *stv1*), *vma3*, *vma11*, *vma16* and *vma6* genes [10]. The V_0_ domain also contains a conserved acidic residue required for proton translocation and a hydrophobic carboxyl domain that is part of the proton pore [9,11]. The V_1_ sector is required for ATP hydrolysis, and the V_0_ sector participates in proton transport [9,12].

V-ATPase is a central player in pH regulation and calcium homeostasis. A previous study demonstrated that loss of V-ATPase function in yeast caused pH-dependent conditional lethality, with mutant strains unable to grow at pH 7 or above but able to grow at pH 5–5.5 [13]. Other fungi, including *Candida albicans*, *Neurospora crassa*, and *Schizosaccharomyces pombe*, also exhibit a pH-dependent growth phenotype when V-ATPase subunits are disrupted [14,15,16]. It has been proposed that *vma* mutants grow at a lower extracellular pH because they can acidify the vacuole via ingestion of acidic extracellular fluid [17]. Furthermore, yeast *vma* mutants are deficient in many aspects of metal ion homeostasis, among which the calcium sensitivity of *vma* mutants is best understood. For example, Kane et al. discovered that *vma* mutants were both sensitive to high levels of extracellular calcium and unable to grow on nonfermentable carbon sources [18]. Elevated calcium caused morphological defects in *vma4-1^ts^* mutants at the nonpermissive temperature [19]. It has also been reported that V-ATPase subunit C is required for the maintenance of calcium homeostasis in *S. cerevisiae* and *C. albicans* [20,21].

Although the combination of pH-dependent growth and calcium sensitivity is a typical result of loss of V-ATPase activity, it should be highlighted that these are far from being the only physiological defects seen in V-ATPase mutants. The fungal cell wall is a crucial organelle that provides a structural barrier, enables biofilm formation, and participates in host-pathogen interactions [22,23]. In *S. cerevisiae*, the main regulatory pathway responsible for maintaining cell wall biosynthesis and responding to cell wall stress is the cell wall integrity (CWI) pathway [24]. Fungi exposed to sublethal concentrations of cell- wall-targeted reagents, such as calcofluor white, Congo Red or caspofungin, exhibit disturbed cell wall biosynthesis and activation of the CWI pathway [25,26]. It has been reported that disruption of *vma*6 or *vph*2 not only leads to weakened hyphal development but also affects cell wall composition and stress resistance in *C. albicans* [27]. In *A. niger*, cell-wall-related genes, including *agsA*, *fksA* and *phiA,* were upregulated in *vma* mutants [28], indicating that the differential expression of these transcription factors may affect the cell wall components of V-ATPase. Taken together, these findings suggest that V-ATPase plays a key role in regulating the CWI pathway.

Among the subunits of V-ATPase, *vma5* encodes subunit C of the V_1_ domain of V-ATPase and is responsible for V_1_ domain assembly in the vacuolar membrane in *S. cerevisiae* [12]. Disruption of *vma5* in *S. cerevisiae* generates a characteristic *vma* deletion phenotype characterized by the inability to grow at high pH and high calcium concentrations [29]. In *C. albicans*, the loss of *vma5* significantly affects filamentous development, vacuole function and calcium homeostasis [21]. Furthermore, in *N. crassa,* the *vma5* mutant shows defects in calcium mobilization [30]. These data indicate that subunit C is essential for the V-ATPase function. However, the functions of yeast VmaC homolog in *A. fumigatus*-VmaC (*Af*VmaC) have never been explored.

In this study, VmaC was characterized and investigated via sequence alignment and functional analysis in *A. fumigatus*. VmaC, putatively encoding subunit C of V-ATPase, was further deleted to uncover its functions during the growth and development of *A. fumigatus*. Our results demonstrated the importance of *AfvmaC* in vacuolar function, maintenance of pH and calcium homeostasis, and regulation of the CWI pathway.

## 2. Materials and Methods

### 2.1. Strains, Media, and Cultural Conditions

All strains used in this study are listed in Table S1 in the supplemental material. Strains were grown in minimal medium (MM) containing 1% glucose, 2% agar, 20× salt solution, and trace elements. MM was supplemented with 5 mM uracil and 10 mM uridine for uracil and uridine auxotroph strains. The liquid glucose MM recipe is identical to that for MM, except without agar. To induce the expression of *vmaC* in the *tet-vmaC* mutant, the medium was supplemented with 1 μg/mL doxycycline. *NiiA-vmaC* strains were grown on MM with 70 mM NaNO_3_ as a nitrogen source, and the *alcA-vmaC* mutant was induced with glycerin as a carbon source. All strains were cultured at 37 °C.

### 2.2. Construction of Strains

Deletions of *vmaC*: the selective marker *pyr4* was amplified from the pAL5 plasmid using the primer pair Pyr4-F/Pyr4-R. Approximately 1 kb of the upstream and downstream flanking sequences of the *vmaC* ORF was amplified with the primer pairs VmaC-P1/P3 and VmaC-P4/P6, respectively. These three PCR products were used as the template to generate the *vmaC* knockout cassette with the primers VmaC-P2/P5. The resulting fusion products were cloned into pEASY-Blunt Zero using a cloning kit (TransGen Biotech, Beijing, China) and used to transform the recipient strain A1160. The transformants were grown on MM and verified by diagnostic PCR using the primers VmaC-diagnostic F/R, VmaC-P1/HPH-R, and VmaC-P6/HPH-F. A similar strategy was used to construct the Δ*vmaA* mutant.

For the construction of complementary *vmaC*, the selected marker *hph* from pAN7-1 was amplified via PCR using the primers HPH-F and HPH-R and then cloned into the pEASY-Blunt vector (TransGen Biotech) to generate the plasmid p-zero-hph. The primers *vmaC*-NotI-F and *vmaC*-NotI-R were used to generate a fragment that included the promoter sequence, the complete ORF, and the 3′UTR of *vmaC*. This fragment was then cloned into the NotI site of the plasmid p-zero-hph and used to transform the Δ*vmaC* deletion strain. For verification of transformants, diagnostic PCR was carried out with the primers *vmaC*-com-up and *vmaC*-com-down.

For the construction of the conditional *tet-vmaC* mutant, the endogenous promoter of *vmaC* was replaced with a conditional doxycycline-inducible Tet-On promoter [31,32].The pyrithiamine resistance cassette and the Tet system from pCH008 were amplified with the primer pair Tet-F/Tet-R. Approximately 1 kb of the upstream and downstream flanking sequences of the *vmaC* promoter regions at positions 802 and +1 were amplified with the primer pairs *vmaC*-conditional-P1/*vmaC*-tet-P3 and *vmaC*-tet-P4/*vmaC*-conditional-P6, respectively. The three purified PCR products were then used as a template to generate the *tet-vmaC* cassette with the primers *vmaC*-conditional-P2/*vmaC*-conditional-P5. The resulting fusion product was cloned into pEASY-Blunt Zero using a cloning kit (TransGen Biotech) and used to transform the WT recipient strain. Transformants were grown on medium supplemented with 0.1 g/mL pyrithiamine (Sigma, St. Louis, MO, USA) and verified by diagnostic PCR using the primer pairs *vmaC*-conditional-P1/tet-ptrA-down and *vmaC*-conditional-P6/tet-ptrA-up. *Tet-vmaC^c^* was constructed by transforming the *tet-vmaC* mutant with the p-zero-hph-*vmaC* plasmid.

For the generation of the *NiiA-vmaC* conditional strain, the *NiiA* fragment was first amplified with the primer pair NiiA-fusion-up/NiiA-down. The primer pairs *vmaC*-conditional-P1/*vmaC*-NiiA-P3 and *vmaC*-NiiA-P4/*vmaC*-conditional-P6 were used to generate the upstream and downstream flanking sequences, respectively. The above-purified PCR products were then used as a template to generate the *NiiA-vmaC* cassette with the primers *vmaC*-conditional-P2/*vmaC*-conditional-P5. Then, the three fusion fragments were cloned into pEASY-Blunt Zero using a cloning kit (TransGen Biotech) and used to transform the WT recipient strain. Diagnostic PCR was performed to verify the transformants using the primer pairs *vmaC*-conditional-P1/NiiA-down and *vmaC*-conditional-P6/NiiA-fusion-up.

For the construction of the conditional *alc-vmaC* mutant, the *alc* fragment was first amplified with the primer pair PyrG+alc-up/PyrG+alc-down. Next, the upstream and downstream flanking sequences of the *vmaC* promoter regions were amplified with the primer pairs *vmaC*-conditional-P1/*vmaC*-alc-P3 and *vmaC*-alc-P4/*vmaC*-conditional-P6, respectively. The three purified PCR products were then used as a template to generate the *alc-vmaC* cassette with the primers *vmaC*-conditional-P2/*vmaC*-conditional-P5. The resulting fusion product was then cloned into pEASY-Blunt Zero using a cloning kit (TransGen Biotech) and used to transform the WT recipient strain. Transformants were verified by diagnostic PCR using the primer pairs *vmaC*-conditional-P1/PyrG+alc-down and *vmaC*-conditional-P6/PyrG+alc-up.

VmaC-GFP was generated as follows: GFP was amplified with the primers GFP+PyrgF and GFP+PyrgR. The upstream and downstream flanking sequences of the *vmaC* promoter regions were amplified with the primer pairs *vmaC*-GFP-P1/*vmaC*-GFP-P3 and *vmaC*-GFP-P4/*vmaC*-GFP-P6, respectively. The three purified PCR products were then used as a template to generate the *vmaC* cassette with the primers *vmaC*-GFP-P2/*vmaC*-GFP-P5. Transformants were verified by diagnostic PCR using the primer pairs GFP-P1/GFP+PyrgR or GFP+PyrgF/*vmaC*-GFP-P6. A similar strategy was used to construct the CccA-RFP strain.

To measure the calcium level in vacuoles, the strain gpd-cpy-Aeq-tet-*vmaC* was generated as follows: PCR was performed using the primers ClaI-cpyA-R and ClaI-cpyA-F to generate a *cpyA’* ORF fragment. The fragment was subcloned into the *Cla*I site of pBARGPE, which has a PgpdA promoter in front of its *Cla*I site, to generate pBARGPE-PgpdA-cpyA. The PgpdA-cpyA fragment was amplified from pBARGPE-PgpdA-cpyA with Ama1-BamHI-gpd-F and CpyA-linker-R primers and then fused with the Aeq-TrpC fragment generated using Linker-Aeq-F and Ama1-BamHI-trpC-R to yield the fusion fragment PgpdA-cpyA-Aeq. The pAMA1-PgpdA-CpyA-Aeq plasmid was generated by ligating PgpdA-cpyA-Aeq into prg3-AMAI-NotI, and then the plasmid was used to transform the recipient strain *Tet-vmaC* to generate the gpd-cpy-Aeq-tet-*vmaC* mutant. A similar strategy was used to construct the gpd-cpy-Aeq-A1160 strain.

To study the ortholog complementation of *A. fumigatus*, the *Tet-vmaC^c-an^ and Tet-vmaC^c-sc^* strains were generated using a similar strategy. In brief, the *vmaC* promoter was amplified using the primers *Af*-vmaC-promoter-p1 and *Af*-*vmaC*-promoter-p3, and the coding sequence was amplified using the primers *An*-*vmaC*-up, *An*-*vmaC*-down, *Sc*-*vmaC*-up and *Sc*-*vmaC*-up. Finally, the above fusion PCR products and the selective marker *pyr4* were cloned into pEASY-Blunt Zero using a cloning kit (TransGen Biotech) and used to transform the recipient strain *Tet-vmaC*.

All the strains and strain annotations are given in Table S1 in Supplementary Data.

### 2.3. Plate Assays

To test the sensitivity of the WT and *Tet-vmaC* strains to cell-wall-perturbing agents, minimal medium was supplemented with 20 μg/mL CFW or 5 μg/mL CR. Then, 2 μL portions of conidial suspensions (1 × 10^7^, 1 × 10^6^, or 1 × 10^5^ conidia/mL) of the indicated strains were spotted on the relevant media plates with or without doxycycline and grown at 37 °C for 48 h for observation and imaging.

### 2.4. RNA Isolation and RT–qPCR

To analyze the relative expression levels of *vmaC* under normal growth conditions, the WT, *Tet-vmaC*, *NiiA-vmaC*, and *alcA-vmaC* strains were incubated in MM for 48 h at 37 °C. To analyze the relative expression of cell wall synthesis genes, WT and the *Tet-vmaC* mutant were incubated in MM for 48 h at 37 °C. To induce the expression of *vmaC* in the *Tet-vmaC* mutant, the medium was supplemented with 1 μg/mL doxycycline. Samples were collected and subsequently frozen using liquid nitrogen. Total RNA was isolated using a UNIQ-10 column total RNA purification kit (Shanghai Sangon Biotech, Shanghai, China) according to the manufacturer’s instructions. For gDNA digestion and cDNA synthesis, a HiScriptII Q RT SuperMix for qPCR (gDNA wiper) kit (Vazyme) was used according to the manufacturer’s instructions. To analyze the relative expression of the genes of interest, the resulting cDNAs were used for quantitative PCR, performed with an ABI one-step fast thermocycler (Applied Biosystems) and AceQ qPCR SYBR green master mix (Vazyme). The results were then normalized to *tubA* expression, and expression levels were calculated using the 2^−ΔΔCT^ method [33].

### 2.5. Western Blotting Analysis

To extract proteins from *A. fumigatus* mycelia, conidia from related strains were incubated in liquid-inducing medium and then shaken at 220 rpm on a rotary shaker at 37 °C for 48 h. The mycelium was ground in liquid nitrogen with a mortar and pestle and suspended in an ice-cold extraction buffer (50 mM HEPES pH 7.4, 137 mM KCl, 10% glycerol, 1 mM EDTA, 1 μg/mL pepstatin A, 1 μg/mL leupeptin, 1 mM PMSF). Equal amounts of proteins (40 μg) per lane were subjected to 10% SDS–PAGE and transferred to PVDF membranes (Immobilon-P, Millipore) in 384 mM glycine, 50 mM Tris (pH 8.4), and 20% methanol at 250 mA for 1.5 h, and the membranes were then blocked with phosphate-buffered saline (PBS), 5% milk, and 0.1% Tween 20. Next, the membrane was probed sequentially with 1:3000 dilutions of an anti-GFP primary antibody (Sigma) and goat anti-rabbit IgG-horseradish peroxidase secondary antibody (Abclonal Co., AS014, Woburn, MA, USA) diluted in PBS, 5% milk, and 0.1% Tween 20. Blots were developed using Clarity ECL western blotting detection reagents (Bio-Rad, Hercules, CA, USA), and images were acquired with a Tanon 4200 Chemiluminescence Imaging System (Tanon, St Andrews, Scotland).

### 2.6. Fluorescence Microscopy

The size of the cover glasses was about 18 × 18 mm (Sangon Biotech, F518211-0001). The medium (1.0 mL) was added gently into the culture dish having the cover glass. For microscopic observation of germlings, fresh conidia of strains in 1.0 mL of liquid MM were grown on sterile glass at 37 °C for 48 h. The resulting hyphae were gently washed with PBS buffer three times and then fixed with 4% paraformaldehyde (Polysciences, Warrington, PA, USA) for 1 h. Then, the cover glasses adhered with germlings were placed upside down in slides for observation and imaging by microscopy. To assess the localization of VmaC-GFP, the mycelia were washed with PBS and then fixed with 4% paraformaldehyde for 40 min at room temperature in the dark. FM4–64 (Sigma–Aldrich, St. Louis, MO, USA) staining was conducted on ice following the manufacturer’s protocol. For CFW staining, hyphae were washed with PBS and stained with 20 μg/mL CFW for ~2 min. All images of the cells were collected with a Zeiss Axio Imager A1 microscope (Zeiss, Jena, Germany).

### 2.7. Transmission Electron Microscopy Analysis of the Cell Wall

The cell walls of the WT and *Tet-vmaC* (Off) strains were examined via TEM as previously described [34]. After the indicated incubation period, the mycelia were fixed overnight in 0.1 M sodium phosphate buffer containing 2.5% glutaraldehyde at 4 °C. The samples were embedded in 1% (wt/vol) agar, fixed in 0.1 M sodium phosphate buffer containing 1% OsO4 for 2 h, and sequentially washed three times in 0.1 M sodium phosphate buffer (15 min each). Next, the samples were dehydrated in 50, 70, 90 and 100% ethanol and 100% acetone for 15 min each. Samples were embedded in 812 epoxy resin monomers (SPI), sliced into 60- to 80-nm ultrathin sections using an ultrathin microtome (Leica UC7), stained with uranyl acetate and lead citrate, and imaged at 80 kV using a transmission electron microscope (Hitachi HT7700, Tokyo, Japan).

### 2.8. Cell Wall Polysaccharide Analysis

WT and Δ*vmaC* strains were incubated in MM for 48 h at 37 °C. After incubation, the mycelia were washed with distilled water and then lyophilized. Fungal cell wall polysaccharides were extracted and quantitatively determined as previously described [35].

### 2.9. Measurement of Intracellular [Ca^2+^]_c_ and Vacuolar Calcium [Ca^2+^]_v_

The strains expressing Aeq/cyp-Aeq were cultured for 2 days at 37 °C to form fresh spores. Fresh spores were filtered through nylon cloth and washed 10 times in distilled deionized water. One million (10^6^) spores in 100 mL liquid MM with/without 2 mM CaCl_2_ were inoculated into each well of a 96-well microtiter plate (Thermo Fisher, Waltham, MA, USA) and incubated at 37 °C for 48 h. The medium was then removed, and the cells in each well were washed twice with PGM (20 mM PIPES pH 6.7, 50 mM glucose, 1 mM MgCl_2_). Aequorin was reconstituted by incubating mycelia in 100 μL PGM containing 2.5 μM coelenterazine f (Sigma–Aldrich, St. Louis, MO, USA) for 4 h at 4 °C in the dark. After the aequorin constitution, mycelia were washed twice with 1 mL PGM and allowed to recover to room temperature for 1 h. Luminescence was measured with an LB 960 Microplate Luminometer (Berthold Technologies, Bad Wildbad, Germany), controlled by a dedicated computer running MikroWin 2000 software. At the 20-s time point of luminescence reading, 0.1 M CaCl_2_ was applied as a stimulant. At the end of each experiment, the active aequorin was completely discharged by permeabilizing the cells with 20% (*v*/*v*) ethanol in the presence of an excess of calcium (3 M CaCl_2_) to determine the total aequorin luminescence in each culture. The conversion of luminescence (relative light units [RLU]) into [Ca^2+^] was performed using Excel 2019 software (Microsoft). Input data were converted using the following empirically derived calibration formula: pCa = 0.332588 (−log k) + 5.5593, where k is luminescence (in RLU) s^−1^/total luminescence (in RLU).

### 2.10. Coimmunoprecipitation and Mass Spectrometry Assay

A GFP antibody (Roche, Basel, Switzerland, 11814460001) was used to pull down VmaC-interacting proteins. A nonlabeled strain under similar conditions was used as a negative and nonspecific VmaC binding control. HPLC was performed at BGI Genomics as a commercial service. In brief, proteins were digested with trypsin (Promega) and labelled using a TMT kit (Thermo Fisher Scientific) according to the manufacturer’s protocol. The labelled tryptic peptides were fractionated via HPLC using a Thermo Betasil C18 column (5 μM particles, 10 mm diameter, 250 mm length). After fractioning, the tryptic peptides were analyzed with an LC-MS/MS system. MS/MS data processing was performed using the MaxQuant search engine (v.1.5.2.8).

### 2.11. Data Analysis

Data are given as the means ± SDs. The SD was obtained from at least three biological replicates. Statistical significance was estimated with Student’s *t*-test using GraphPad Prism 7 software. *p* values less than 0.05 were considered statistically significant.

## 3. Results

### 3.1. Fungal Conserved VmaC Is Important for the Growth and Conidiation of A. fumigatus

**Figure 1:**
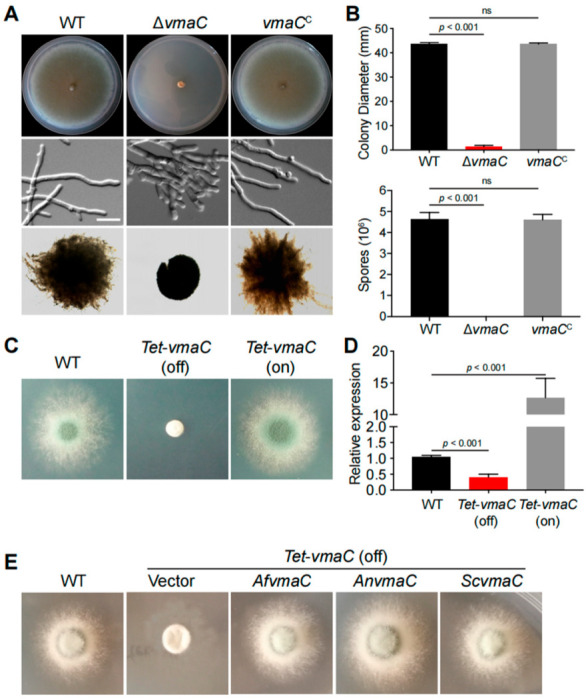
Phenotypes of the indicated *vmaC* mutants in solid and liquid minimal media. (**A**) Colony morphology of the Δ*vmaC* and reference strains cultured on solid and liquid minimal media at 37 °C. (**B**) Colony diameter and quantitative total conidial production for the indicated strains are shown in Panel A. Values represent the mean ± SD of three replicates (ns, not significant). (**C**) Colony morphology of the conditional *vmaC* mutants cultured on noninducing medium (Tet-off) and inducing medium (Tet-on) at 37 °C for 2 days. (**D**) The transcript levels in the *tet-vmaC* conditional strains were grown at 37 °C for 2 days. Values represent the mean ± SD of three replicates. (**E**) Complementation experiments in which the conditional *vmaC*-turned-off strain was transformed with full-length cDNA from *A. nidulans* and *S. cerevisiae* under the control of the native *AfvmaC* gene promoter. *Af*, *A. fumigatus*; *An*, *A. nidulans*; *Sc*, *S. cerevisiae*.

We found a putative *S. cerevisiae* VmaC homolog, *Af*VmaC (AFUB_010230), in *A. fumigatus* using the *S. cerevisiae* VmaC as a query in BLASTp analysis, which showed an identity of 35.86% between *Sc*VmaC and *Af*VmaC (Figure S1A). To explore the functions of the *vmaC* gene in *A. fumigatus*, we first constructed a *vmaC* full deletion strain via homologous gene replacement with the *N. crassa pyr4* marker. Diagnostic PCR analysis showed that the homologous replacement was successful and that *vmaC* was truly deleted in this *vmaC* deletion strain (Figure S2). As shown in Figure 1A,B, Δ*vmaC* showed severely sick colony phenotypes with reduced radial growth and no conidia production compared to the parental wild-type (WT) strain when cultured on solid minimal medium. Consistently, under liquid culture conditions, the Δ*vmaC* mutant exhibited branched hyphal growth and very small mycelial balls with smooth margins, compared to the parental WT strain, suggesting that loss of *vmaC* results in defective hyphal tip growth with a reduced growth rate. To confirm that these phenotypes were specifically due to the lack of *vmaC*, a full-length *vmaC* gene was introduced into the Δ*vmaC* mutant. As shown in Figure 1A, all the above-tested phenotypes were restored to the status of the WT strain under both solid and liquid culture conditions, indicating that VmaC is required for hyphal growth and conidiation.

To further explore the relationship between *vmaC* expression and its function, we subsequently generated *niiA::vmaC* and *alcA::vmaC* conditional strains but failed to obtain any functional mutants (Figure S3). Therefore, we constructed a conditional *tet*-*vmaC* strain under the control of the Tet on-off system. As shown in Figure 1C,D, the *tet-vmaC* conditional strain displayed the same phenotype as the Δ*vmaC* mutant when growing on noninducing medium (Tet-off). On the inducing medium (Tet-on), a colony phenotype similar to that of the WT strain was observed. As predicted, the *vmaC* mRNA level was normal in the corresponding media, indicating that the conditional promotor worked normally. Thus, collectively, these results demonstrate that VmaC is required for hyphal growth and conidiation in *A. fumigatus*. However, it seems that VmaC is not essential because deletion of *vmaC* or turn-off of *vmaC* expression was not lethal, leading only to very sick colonies for the related strains.

To further investigate whether the fungal VmaC orthologs between yeast and *Aspergillus* are functionally conserved, we performed BLAST research using the *Af*VmaC amino acid sequence and identified the orthologues in fungal species. Subsequently, we implemented complementation experiments by transforming the conditional *vmaC*-turned-off strain with the full-length cDNA of *A. nidulans* and *S. cerevisiae* under the control of the native *AfvmaC* gene promoter. As shown in Figure 1E, colonies transformed with *A. nidulans* and *S. cerevisiae vmaC* cDNA showed similar colony phenotypes with remarkable hyphal growth, which was similar to that of the WT strain. In contrast, the strain transformed by the vector still showed very sick colonies (Figure 1E). These data suggest that the *A. nidulans* and *S. cerevisiae* VmaC orthologs can act as functional substitutes for VmaC in *A. fumigatus* colony growth.

### 3.2. Molecular Characterization of Vacuole-Localized VmaC

**Figure 2:**
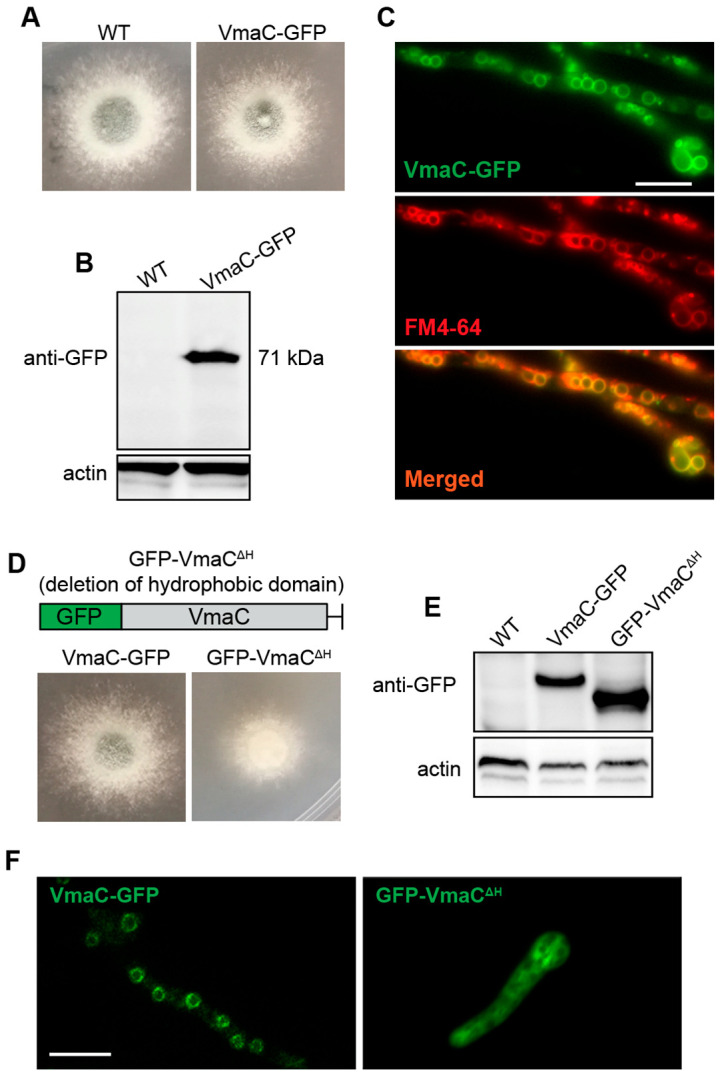
VmaC is localized in the vacuole. (**A**,**D**) Colony morphology of the VmaC-GFP strain and the VmaC C-terminal hydrophobic domain truncation mutant (VmaC^ΔH^) after culture at 37 °C for 2 days. (**B**,**E**) Western blot analysis using an anti-GFP antibody indicated that the VmaC-GFP fusion protein was present and showed a predicted size of approximately 71 kDa. The VmaC C-terminal hydrophobic domain truncation mutant GFP-VmaC^ΔH^ showed the predicted truncated VmaC band. Actin was used as an internal loading control. (**C**) Colocalization of the C-terminal VmaC-GFP truncation strain with FM4-64 staining. The yellow color in the merged image indicates colocalization. (**F**) Localization in the VmaC-GFP and GFP-VmaC^ΔH^ strains. Bar, 5 μm.

To ascertain the subcellular localization of VmaC during growth, we labelled the C-terminus of VmaC with a GFP tag under the native VmaC promoter. After constructing the labelled strain, we first tested whether the VmaC-GFP strain was functional. As shown in Figure 2A, the colony phenotype of the VmaC-GFP strain was similar to that of the WT strain, suggesting that VmaC-GFP is functional and that GFP labelling did not affect the function of VmaC. Therefore, we next examined the signal of VmaC-GFP using fluorescence microscopy. As shown in Figure 2C, VmaC-GFP showed a very clear vacuolar localization pattern in hyphal cells. We then used the vacuolar endomembrane marker FM4-64 to stain the VmaC-GFP strain. As shown in the merged image in Figure 2C, the majority of VmaC-GFP colocalized with FM4-64. Moreover, western blotting analysis with an anti-GFP antibody showed a band at approximately 71 kDa (including the 27 kDa of GFP) (Figure 2B), indicating that the molecular weight of VmaC is approximately 44 kDa, which is consistent with the predicted size. Next, we wondered whether the C-terminal hydrophobic domain was required for the localization and function of VmaC. Using VmaC-tagged GFP, we constructed a VmaC truncation mutant that lacked a VmaC C-terminal hydrophobic domain. Notably, the VmaC C-terminal hydrophobic domain truncation mutant (VmaC^ΔH^) showed a colony phenotype similar to that of the full-length deletion mutant (Δ*vmaC*), with very sick hyphal growth in both solid and liquid minimal media (Figure 2D), suggesting that the C-terminal hydrophobic domain is required for the function of VmaC. Nevertheless, a VmaC-H truncated protein band could not be detected via western blotting, suggesting that the C-terminal hydrophobic domain is required for the normal localization and expression of VmaC. Next, we generated another GFP-tagged VmaC truncation strain by fusing GFP at the N-terminus of VmaC. In the western blotting assay, GFP-VmaC^ΔH^ showed the predicted truncated VmaC band (Figure 2E). However, the majority of the GFP-truncated VmaC was localized in the cytoplasm (Figure 2F), indicating that the C-terminal hydrophobic domain of VmaC plays an important role in normal VmaC vacuolar localization.

### 3.3. VmaC Participates in the Maintenance of Calcium Homeostasis and Vacuolar Acidity

**Figure 3:**
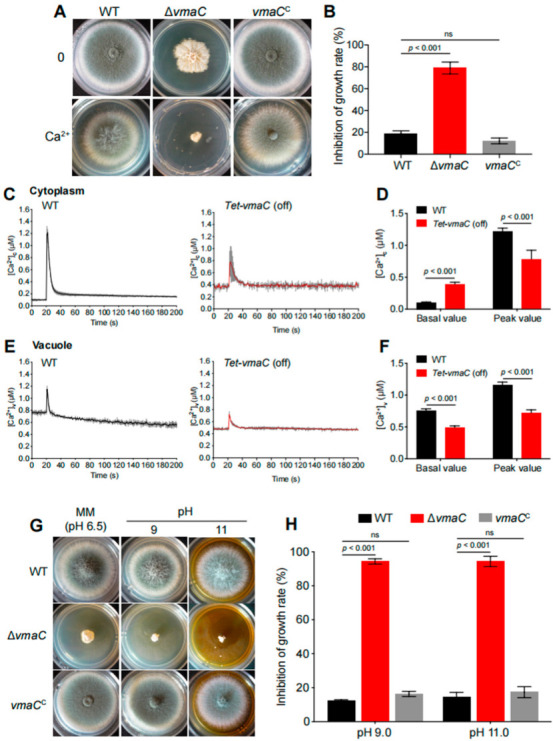
High calcium concentrations and high pH are toxic to *vmaC* mutants. (**A**) Colony morphology of Δ*vmaC* in response to an extracellular calcium stimulus at 37 °C for 2 days. (**B**) The growth inhibition rate of Δ*vmaC* when treated with 500 mM calcium (ns, not significant). (**C**) Linear graphs indicating the real-time [Ca^2+^]_c_ changes in response to calcium stimulus. [Ca^2+^]_c_, the free Ca^2+^ concentration in the cytosol. (**D**) The comparison of [Ca^2+^]_c_ of WT and *Tet-vmaC* (off) in resting and dynamic levels. The basal value is the resting level prior to the extracellular calcium stimulus. The peak value is the peak level after the extracellular calcium stimulus. Data are the average of three experiments. Error bars show the standard deviation. Statistical significance was determined by Student’s t-test. (**E**) Linear graphs indicating the real-time [Ca^2+^]_v_ changes in response to calcium stimulus. [Ca^2+^]_v_, the free Ca^2+^ concentration in vacuoles. (**F**) The comparison of [Ca^2+^]_v_ of WT and *Tet-vmaC* (off) in resting and dynamic levels. (**G**) The morphology of mycelial pellets of the WT and Δ*vmaC* strains under pH = 4, pH = 9, and pH = 11 conditions. (**H**) The growth inhibition rate of Δ*vmaC* when treated with pH 4, pH 9, and pH 11, respectively (ns, not significant).

The aforementioned data indicate that VmaC was localized in the vacuole and that the vacuole is a major intracellular Ca^2+^ store involved in the regulation of Ca^2+^ homeostasis [36]. We examined whether the Δ*vmaC* mutant in *A. fumigatus* was sensitive to Ca^2+^ cations. As shown in Figure 3A,B, the Δ*vmaC* mutant was hypersensitive to high concentrations of calcium. We also found that the *vmaC* mutant showed no phenotype for the sensitivity in Mg, Cd, Cu, Co, and Mn stimuli (Figure S4), indicating that VmaC may not affect pathways related to these cations. These results suggest that VmaC may be involved in the regulation of calcium homeostasis in cells. We then monitored the changes in cytoplasmic intracellular [Ca^2+^]_c_ and vacuolar [Ca^2+^]_v_ calcium levels in living cells of the WT and conditional *vmaC*-turned-off strains that were transformed with cytoplasmic aequorin and vacuolar aequorin, respectively. As shown in Figure 3C, after treatment with 0.1 M CaCl_2_, the [Ca^2+^]_c_ concentration in WT cells transiently increased from a resting level of approximately 0.1 μM to a peak of 1.1 μM. In comparison, the *vmaC* conditional strain had a resting level of 0.45 μM, and then transiently increased to 0.7 μM. These data indicate that the turn-off of *vmaC* expression caused a markedly increased resting [Ca^2+^]_c_ level (0.1 μM increased to 0.45 μM) but a reduced calcium transient peak (1.1 μM reduced to 0.7 μM). However, for [Ca^2+^]_v_, the turn-off of *vmaC* expression resulted in a decreased basal value (from 0.7 μM to 0.45 μM) and vacuolar peak value (from 1.15 μM to 0.7 μM). These data suggest that *vmaC* may be involved in calcium transportation from the cytoplasm to the vacuole and that deficiency of VmaC induces abnormal cytoplasmic calcium accumulation. We then inoculated the *ΔvmaC* mutant on different pH minimal media and found that the *ΔvmaC* mutant was sensitive to high pH (Figure 3G,H). Together, these results suggest that the VmaC is required for vacuolar function.

### 3.4. VmaC Is a Subunit of the V-ATPase Complex

In yeast, previous studies have demonstrated that the *vmaC* gene encodes subunit C, which is a V1 sector subunit that connects the V1 sector with the V0 sector of the enzyme [12]. We wondered if VmaC in *A. fumigatus* is also a subunit of V-ATPase that interacts with other subunits localized in the vacuole. To test this hypothesis, we carried out a coimmunoprecipitation combined with mass spectrometry assay to detect the VmaC-interacting complex. We first constructed a GFP-labeled C-terminus in the VmaC strain, and then, a GFP antibody was used to pull down the VmaC-interacting complex for proteomics-based assays. A nonlabelled strain under similar conditions was used as a negative and nonspecific VmaC binding control. As shown in Table S1, 281 proteins were identified as potential VmaC-interacting proteins in the VmaC::GFP strain, but they were absent in the non-VmaC-GFP-labeled strain. Among these putative VmaC-interacting proteins, VmaA, VmaB, VmaE, VmaH, and VmaC were identified, all of which are subunits of V-ATPase (Table 1), suggesting that *Af*VmaC is a subunit of the V-ATPase complex. In addition, *Af*VmaC had the ability to bind itself. Moreover, we found that cell wall biosynthesis-related proteins were present in the list of putative VmaC-interacting proteins, including RlmA, ChsE, Fks1 and Lkh1, suggesting that VmaC may affect cell wall biosynthesis.

**Figure 4:**
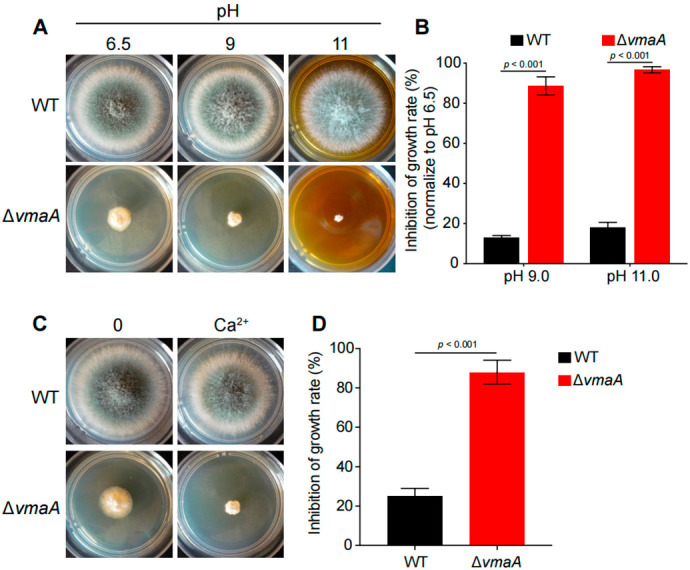
VmaC is a subunit of the V-ATPase complex. (**A**) The morphology of mycelial pellets of the wild-type and Δ*vmaA* strains under different pH 6.5, pH 9, and pH 11 conditions. (**B**) The growth inhibition rates of Δ*vmaA* when treated with pH 9 and pH 11. (**C**) The morphology of mycelial pellets of the wild-type and the Δ*vmaA* strains in 500 mM calcium stimuli. (**D**) The growth inhibition rates of Δ*vmaA* when treated with 500 mM calcium.

To further confirm whether these putative pull-down subunits of the V-ATPase complex have functions similar to that of VmaC, we constructed another subunit deletion of the V-ATPase complex—the Δ*vmaA* mutant (the VmaA subunit is a major contributor to V-ATPase activity). As shown in Figure 4, the Δ*vmaA* mutant showed severely reduced radial growth and no conidia production, similar to the phenotype of the Δ*vmaC* mutant when inoculated in the same minimal medium. Both deletion strains showed lethality at high calcium concentrations and high pH. These results demonstrate that the V-ATPase activity contributed by the Vma complex plays an important role in hyphal growth and conidiation.

### 3.5. AfVmaC Is Involved in the Regulation of the Cell Wall Integration Pathway

**Figure 5:**
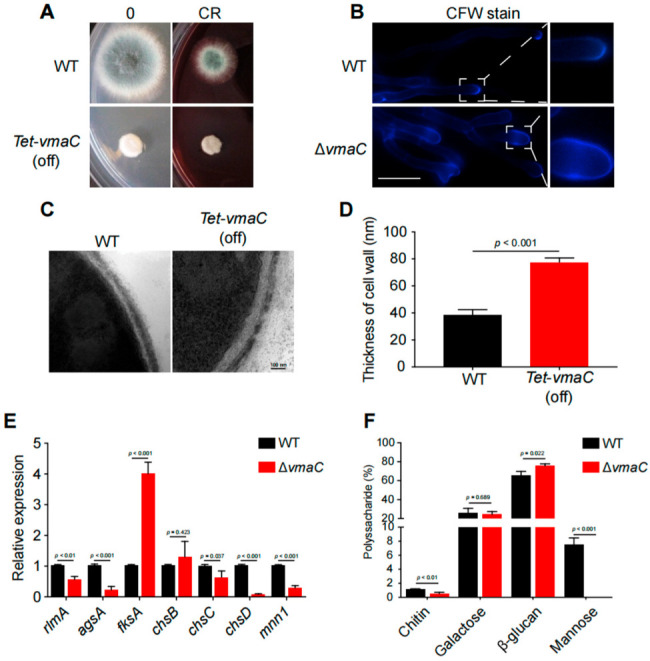
VmaC regulates cell wall architecture and composition. (**A**) The morphology of mycelial pellets of the WT and Δ*vmaC* strains cultured on minimal medium supplemented with or without 75 μg/mL CR. (**B**) Staining of the WT and Δ*vmaC* strains for chitin with CFW. Scale bar, 10 μm. (**C**) Representative TEM images of hyphae of WT and *tet*-*vmaC* (off) strains cultured on minimal medium. Scale bar, 100 nm. (**D**) Quantification of the mean cell wall thickness of the WT and *tet*-*vmaC* (off) strains as in Panel C. The data are presented as the means and standard deviations of three biological samples, with 10 sections measured for each experimental group. (**E**) Quantitative analysis of the expression of cell-wall-related genes. All the results were obtained in three independent biological experiments (ns, not significant). (**F**) Absolute polysaccharide composition of the mycelial cell walls in WT and *tet*-*vmaC* (off) strains. The data are presented as the means and standard deviations of three biological replicates (ns, not significant).

Since the above data suggest that many cell wall biosynthesis-related proteins are associated with the *Af*VmaC-interacting complex, we wondered whether *Af*VmaC deficiency could influence cell wall biosynthesis. Unexpectedly, compared to the WT strain, the conditional *vmaC*-turned-off mutants showed high tolerance to the cell-wall-stress reagent Congo Red (CR) (Figure 5A), suggesting that VmaC may be involved in the CWI pathway. To further verify this possibility, the nonspecific fluorochrome calcofluor white (CFW), which binds to cellulose and chitin cell walls, was used to stain the Δ*vmaC* mutant and its parental WT strain. As shown in Figure 5B, every hyphal tip had an accumulated CFW stain in the WT strain. However, in the Δ*vmaC* mutant, there was no clear CFW stain in the hyphal tips; instead, CFW was distributed in the subtip of hyphal cells. These data suggest that the chitin distribution in hyphal cells was disordered in the Δ*vmaC* mutant. Next, the thickness of the cell wall was detected using transmission electron microscopy (TEM). The data indicated that the conditional *vmaC*-turned-off strain displayed thicker cell walls (approximately 2-fold) than the WT strain (Figure 5C,D). To further explore how the cell wall architecture was changed, the mRNA expression levels of genes related to the CWI pathway were analyzed. As shown in Figure 5F, the cell wall biosynthesis-related transcription factor *rlmA*, α-1,3-glucan synthase-encoding gene *agsA*, chitin synthase-encoding genes *chsC* and *chsD*, and α-1,6-mannosyltransferase encoding gene *mnn1* were significantly decreased in the Δ*vmaC* mutant. In comparison, *fks1* (β-1,3-glucan synthase) showed enhanced expression in the Δ*vmaC* mutant (Figure 5F). These data suggest that *vmaC* may be involved in the expression of cell wall biosynthesis-related genes and thereby affect cell wall components. To quantify the cell wall components, a high-performance liquid chromatography (HPLC) assay was used to detect monosaccharide ratios for polysaccharide components of cell walls. As shown in Figure 5E, the results showed that chitin and mannose were decreased and that galactose and β-glucan were increased in the Δ*vmaC* mutant. Collectively, these data demonstrated that loss of VmaC results in abnormal cell wall architecture and changes in cell wall components.

## 4. Discussion

V-ATPase is a multisubunit complex that is involved in a variety of cellular processes [6]. In this study, we functionally characterized the roles of an evolutionarily conserved subunit of the V-ATPase complex, VmaC, in *A. fumigatus*. According to the sequence alignments and functional analysis, we identified *AfvmaC* as a subunit of the V-ATPase complex. Both the *vmaC* deletion and the *vmaC*-turned-off mutants resulted in phenotypic defects in *A. fumigatus* hyphal growth and conidiation, which highlights the importance of VmaC in these biological processes. Complementation experiments between different fungal species verified that the homologous VmaC of *S. cerevisiae* could rescue the sick phenotype of the *tet-vmaC* (off) conditional strain (Figure 1E), indicating that VmaC functions as a subunit of V-ATPase and its functions are relatively conserved. In yeast, cells appear to be able to tolerate the loss of V-ATPase function but with conditional defective growth and morphological phenotypes [37]. However, in *A. fumigatus*, deletion of *AfvmaC* or turn-off of *AfvmaC* expression was not lethal but led to a very sick and tiny colony phenotype in all tested media, which is different from the conditional defects caused by deletion of *vmaC* in yeast. This suggests that although VmaC functions are conserved among various fungal species, the roles of important functional domains may differ. In addition, disruption of the C-terminal hydrophobic domain of VmaC in *A. fumigatus* not only resulted in a colony phenotype similar to that of the *vmaC* deletion mutant but also led to mislocalization of VmaC, indicating that the C-terminal hydrophobic domain of VmaC was needed for the function and proper distribution of VmaC. As a storage organelle, vacuoles are associated with many aspects of cellular homeostasis [5]; thus, vacuole-localized VmaC in *A. fumigatus* may be involved in the normal functions of vacuoles. Defects in VmaC-involved V-ATPase activity may result in an inability to store or exchange nutrients between vacuoles and the cytosol during germination.

Calcium is vital for cells to translate diverse developmental cues and environmental stresses into specific cellular compartment developmental responses, and V-ATPase plays an important role in the maintenance of ion homeostasis in eukaryotes [38]. It has been reported that cytosolic calcium homeostasis is a constitutive function of the V-ATPase in *S. cerevisiae* [39]. However, due to an inability to examine the dynamic vacuolar calcium concentration, there was no direct evidence showing a relationship between VmaC and calcium homeostasis before this study. Here, through an intracellular calcium dynamic monitoring strategy, we discovered that VmaC dysfunction disturbed calcium homeostasis in both the cytosol and vacuole, resulting in significant cell growth inhibition in the presence of a calcium stimulus. Typically, the resting [Ca^2+^]_c_ in *A. fumigatus* ranges from 0.1 to 1.1 μM. In the *vmaC*-turned-off mutant, the cytosolic resting calcium level increased by 4.5-fold compared to that in the WT strain before the calcium stimulus. However, when cells were challenged with a high calcium concentration, the Δ*vmaC* mutant displayed a 16% decrease in the [Ca^2+^]_c_ amplitude compared to the WT strain. The sustained increase in [Ca^2+^]_c_ led us to consider whether calcium homeostasis in the vacuole would also be affected by a lack of VmaC. Surprisingly, we found that the basal resting [Ca^2+^]_v_ and the transient [Ca^2+^]_v_ in the *vmaC*-turned-off mutant were significantly increased in the presence of an extracellular calcium stimulus. Collectively, these data demonstrated that VmaC not only regulates resting cytosolic calcium but also affects the vacuolar calcium transient response in *A. fumigatus*. Because of the evolutionarily conserved role of VmaC, we suggest that VmaC may also participate in maintaining cytosolic calcium homeostasis in other fungal species. In addition to disordered intracellular calcium homeostasis, the Δ*vmaC* mutant exhibited hypersensitivity to high pH, which is in accord with the Δ*vmaA* mutant. Since V-ATPase is involved in the maintenance of intracellular pH homeostasis through its role in pumping excess protons into the vacuole [6], we speculate that VmaC is involved in the reciprocal transport of Ca^2+^ and H^+^ in *A. fumigatus*. Defects in pH and/or calcium homeostasis could potentially account for both the cytoskeletal defects and the morphological changes in the Δ*vmaC* mutant [40,41,42].

Furthermore, the conditional *vmaC*-turned-off mutants were tolerant of the cell wall stress reagent CR, and GFP-trap data further revealed that VmaC may interact with cell wall biosynthesis-related proteins (Figure 2B), indicating that VmaC may play a central role in the cell wall biosynthesis pathway. Since proper V-ATPase function affects multiple cellular processes, several scenarios can be envisioned to explain this phenomenon. One scenario is that the loss of VmaC affects the transport of secretory vesicles to the tip of the fungal cell (Figure 5B). These vesicles contain the enzymes that are needed for proper cell wall biosynthesis, and it is conceivable that less efficient secretion leads to improper cell wall biosynthesis. Another possibility is that the *vmaC* mutant triggered the CWI pathway via regulation of related transcription factors (Figure 5E). Accordingly, we found that the cell wall of the conditional *vmaC*-turned-off mutant was significantly thicker than that of the wild-type strain. In addition, qRT–PCR analysis showed that the transcription factor *fksA* was significantly upregulated in the Δ*vmaC* mutant, in accord with findings in *A. niger* [28], while other transcription factors (e.g., *rlmA*, *adsA*, *chsC*, *chsD*, and *mnn1*) were downregulated, suggesting that VmaC plays a central role in regulating cell wall components. We demonstrated that the chitin and mannose components of cell walls were decreased in the Δ*vmaC* mutant, and galactose and β-glucan were increased compared to levels in the parental WT, further indicating that the functions of VmaC are related to the CWI pathway. In addition, azoles function by inhibiting ergosterol synthesis, while ergosterol is a critical requirement in V-ATPase function. The mutants defective in ergosterol biosynthesis exhibited most of these characteristic *vma* deletion phenotypes [43]. Notably, deletion of *vmaC* leads to disorders in vacuolar localization and function [43]. In both *S. cerevisiae* and *C. albicans*, fluconazole impaired vacuolar acidification, whereas concomitant ergosterol feeding restored V-ATPase function and cell growth [43]. These findings suggest that the critical requirement for ergosterol in V-ATPase function may underlie the antifungal activity of azoles. Although there is no direct evidence showing that *vma* mutants are related to drug resistance in *A. fumigatus*, with the high conservation of VmaC, the role of VmaC in drug resistance may exist in *A. fumigatus* as well. This is an interesting research area which can be addressed in the near future.

Taken together, to the best of our knowledge, this is the first report in which the function of the VmaC subunit of the V-ATPase complex has been identified in *A. fumigatus*. Our study reveals that VmaC is essential for hyphal growth and conidiation in *A. fumigatus*. Notably, deletion of *vmaC* leads to disorders in vacuolar localization and function, probably by affecting calcium homeostasis. Furthermore, *vmaC* deletion increases the tolerance to cell-wall-stress reagents and regulates the cell wall pathway in *A. fumigatus*. Therefore, our findings enrich the understanding of V-ATPase and may provide a potential antifungal target.

## Figures and Tables

**Table 1:** Selected Members of VmaC interacting proteins.

Systematic Name	Description
	**V-ATPase subunits**
AFUB_050900	Vacuolar ATP synthase catalytic subunit A
AFUB_028870	Vacuolar ATP synthase subunit B
AFUB_096250	Vacuolar ATP synthase subunit E
AFUB_010100	Vacuolar ATP synthase subunit H
AFUB_093460	Vacuolar ATP synthase subunit C
	**Cell Wall Biosynthesis**
AFUB_059750	Alpha-1,2-mannosyltransferase (Kre5)
AFUB_078400	1,3-beta-glucan synthase catalytic subunit Fks1
AFUB_079180	Mutanase AgnE
AFUB_029080	Chitin synthase ChsE
AFUB_097510	Alpha-1,3-glucanase/mutanase
AFUB_040580	SRF-type transcription factor RlmA
AFUB_041400	Alpha-1,3-glucosyltransferase
AFUB_048110	Mannan endo-1,6-alpha-mannosidase
	**Protein kinase**
AFUB_007300	Serine/threonine protein kinase
AFUB_098900	Protein kinase activator Bem1
AFUB_016170	Protein kinase Lkh1
AFUB_005320	Serine/threonine-protein phosphatase
AFUB_051670	Protein kinase

## Data Availability

All data are publicly available.

## Supplementary Materials

The following supporting information can be downloaded at: https://www.mdpi.com/article/10.3390/jof8111219/s1, Figure S1: Phylogenetic relationships of the homologs of VmaC; Figure S2: PCR results demonstrate that vmaC is successfully deleted; Figure S3: The construction of conditional stains and the measurement of vmaC expression level; Figure S4: The effect of different metal ions on the vmaC deletion mutants; Figure S5: The original nucleic acid gel electrophoresis images; Table S1: The list of 281 potential VmaC interacting proteins; Table S2: Strains used in this study; Table S3: Primers used in this study.
