# Supplementary material for: Subunit C of V-ATPase-VmaC Is Required for Hyphal Growth and Conidiation in A. fumigatus by Affecting Vacuolar Calcium Homeostasis and Cell Wall Integration
Source: J Fungi (Basel). 2022 Nov 17;8(11):1219. doi: 10.3390/jof8111219 (PMC9699406; doi:10.3390/jof8111219)
Supplement: Supplementary file 1 [file jof-08-01219-s001.zip › Fig S5.pdf]

Figure S4: The original gel electrophoresis images.

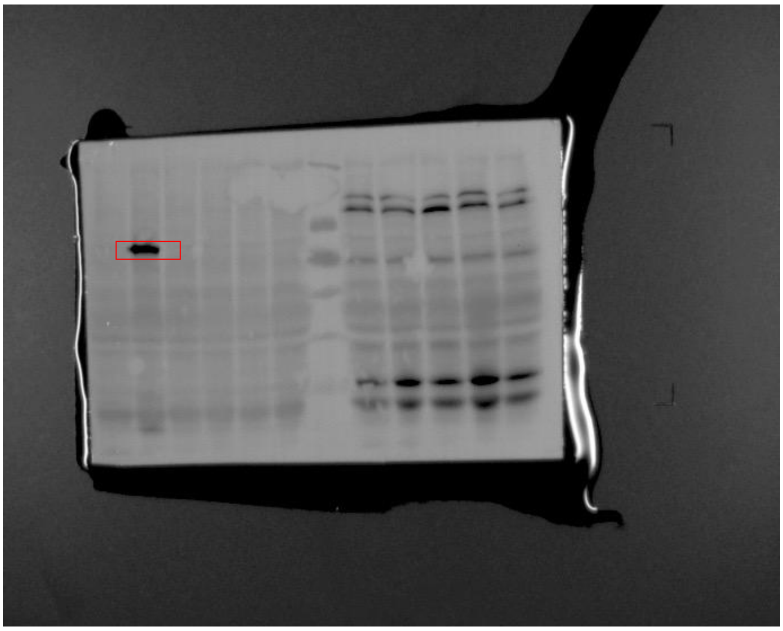

(A) VmaC-GFP (red box) from Fig. 2B.

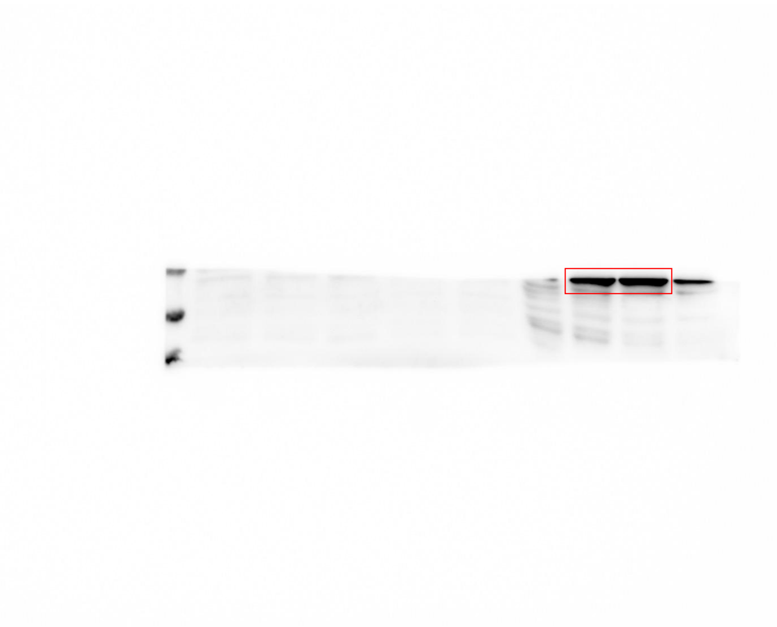

(B) Actin-GFP (red box) from Fig. 2B.

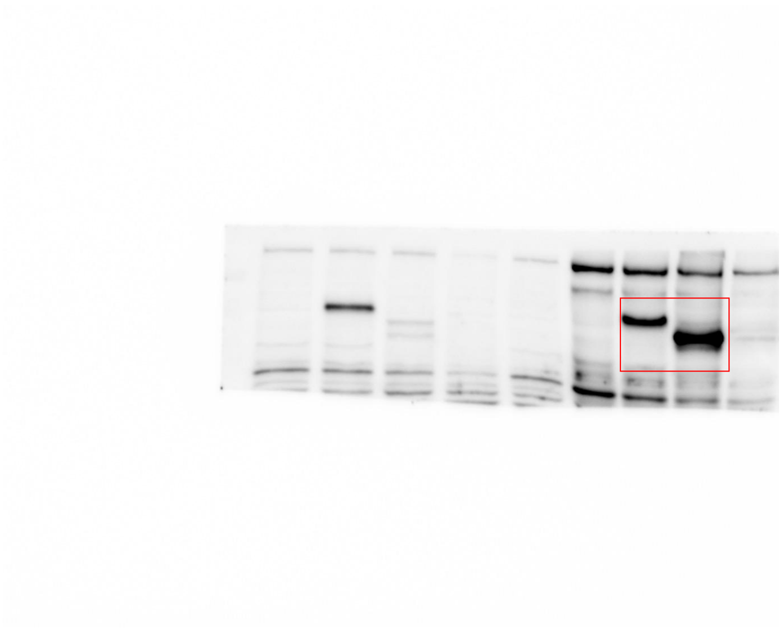

(C) VmaC-GFP (red box in the left) and GFP-VmaC$^{\Delta H}$ (red box in the right) from Fig. 2D.

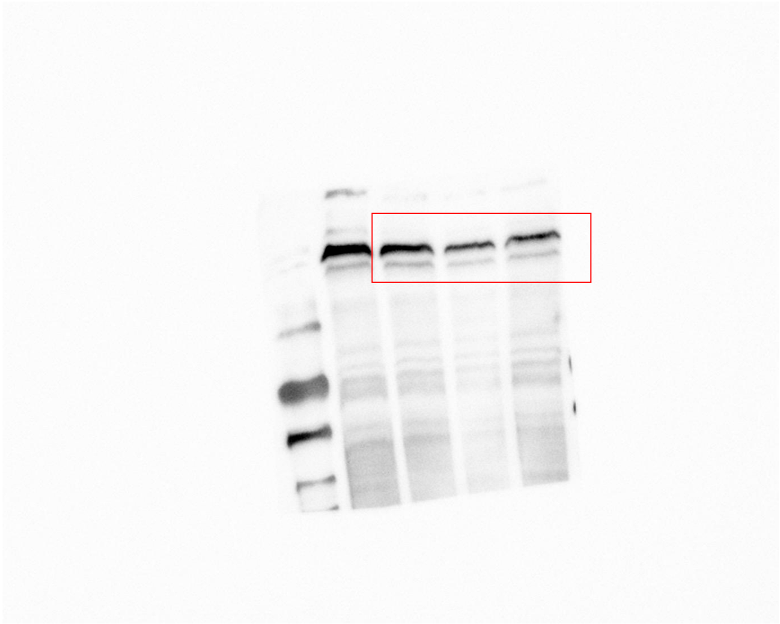

(D) Actin (red box) from Fig. 2D.

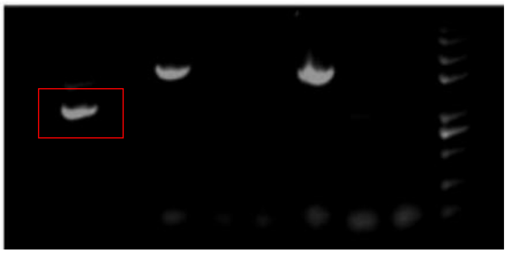

(E) WT (red box) from Fig. S2B.

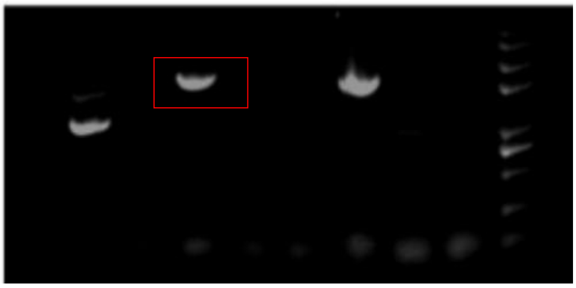

(F) *ΔvmaC* (red box) from Fig. S2B.
