# Supplementary material for: Subunit C of V-ATPase-VmaC Is Required for Hyphal Growth and Conidiation in A. fumigatus by Affecting Vacuolar Calcium Homeostasis and Cell Wall Integration
Source: J Fungi (Basel). 2022 Nov 17;8(11):1219. doi: 10.3390/jof8111219 (PMC9699406; doi:10.3390/jof8111219)
Supplement: Supplementary file 1 [file jof-08-01219-s001.zip › Table S2.pdf]

S2 Table. Strains used in this study

Strain name	Mutated genes	Genotype	Source
A1160	Parental strain	*Δku80; pyrG1*	FGSC
SCC1	*△vmaC::hph*	*△Ku80; pyrG;△vmaC::hph*	This work
SCC2	△vmaC::hph;vmaC::pyr4	*△Ku80; pyrG;△vmaC::hph;vmaC::pyr4*	This work
SCC4	niiA(P)-vmaC::pyr4	*△Ku80; pyrG;niiA(P)-vmaC::pyr4*	This work
SCC5	alc(P)-vmaC::pyrG	*△Ku80; pyrG;alc(P)-vmaC::pyrG*	This work
SCC6	tet(P)-vmaC::ptrA	*△Ku80; pyrG;tet(P)-vmaC::ptrA*	This work
SCC7	vmaC::GFP::pyrG	*△Ku80; pyrG;vmaC::GFP::pyrG*	This work
SCC8	vmaC::GFP::pyrG;cccA::RFP;sef::hph	*△Ku80;pyrG;vmaC::GFP::pyrG;cccA::RFP;sef::hph*	This work
SCC11	gpd-cpy-pAEQ-aeqs::pyr4	*△Ku80; pyrG;gpd-cpy-pAEQ-aeqs::pyr4*	This work
SCC12	tet(P)-vmaC::ptrA;gpd-cpy-pAEQ-aeqs::pyr4	*△Ku80; pyrG;tet(P)-vmaC::ptrA;gpd-cpy-pAEQ-aeqs::pyr4*	This work
SCC13	△vmaA::pyr4	*△Ku80; pyrG;△vmaA::pyr4*	This work
SCC14	*tet(P)-vmaC::ptrA;AfvmaC::pyr4*	*△Ku80; pyrG;tet(P)-vmaC::ptrA;AfvmaC::pyr4*	This work
SCC15	tet(P)-vmaC::ptrA;AnvmaC::pyr4	*△Ku80; pyrG;tet(P)-vmaC::ptrA;AnvmaC::pyr4*	This work
SCC16	tet(P)-vmaC::ptrA;ScvmaC::pyr4	*△Ku80; pyrG;tet(P)-vmaC::ptrA;ScvmaC::pyr4*	This work
