# Supplementary material for: Subunit C of V-ATPase-VmaC Is Required for Hyphal Growth and Conidiation in A. fumigatus by Affecting Vacuolar Calcium Homeostasis and Cell Wall Integration
Source: J Fungi (Basel). 2022 Nov 17;8(11):1219. doi: 10.3390/jof8111219 (PMC9699406; doi:10.3390/jof8111219)
Supplement: Supplementary file 1 [file jof-08-01219-s001.zip › Table S3.pdf]

S3 Table. Primers used in this study

Name	Sequence (5' to 3')	Purpose
vmaC-p1	GTCTGCCTACAAAGTCCCA	Fusion PCR for *vmaC* knockout
vmaC-p2	AATCGGACGCTCCCGCCTT	
vmaC-p3	GCCTGTGTGTAGAGATACAAGGGAATTCGA TGTCGGGACGCGATGCAGG	
vmaC-p4	TAAGCGCCCACTCCACATCTCCACTCGATG GCGGGATGATGCACTACGC	
vmaC-p5	GAATGTAAACTGACAGGAG	
vmaC-p6	GACCCAAAGCGTTCAGTC	
HPH-F	GAATTCCCTTGTATCTCTACACACAGGC	
HPH-R	TCGAGTGGAGATGTGGAGTGGGCGCTTA	
vmaC-diag-F	ATCACTCCCTCCCACCAC	*vmaC* knockout confirmation
vmaC-diag-R	AACAAACACCCGCAGAAC	
vmaC-com-up	GAGCCTGACTGCCATCTT	Complementary *vmaC* construction
vmaC-com-do wn	GTAACGCCAGGGTTTTCCCTCGGGTAATCC ACCAT	
Pyr4-up	GGAAAACCCTGGCGTTAC	
Pyr4-down	CTATGCGGCATCAGAGCA	
vmaC-com-up-diag	CTGCTCGCTGACTGAACC	Complementary *vmaC* construction confirmation
vmaC-com-do wn-diag	CTTCCTCCCATCCGTGTC	
vmaC-conditio nal-p1	CCAGTCCCGTCCAGAGCTTT	Fusion PCR for *vmaC* conditional strain
vmaC-conditio nal-p2	CGAAGTCTCCACTAAGCAC	
vmaC-conditio nal-p5	GCGGAGTCAACATACCCT	
vmaC-conditio nal-p6	CTCGTAGTACACATACGCAG	
vmaC-*tet*-p3	GACAGTATAATACAAACAAAGATGCAAGATG TGAATCAATTATGCAGT	*Tet-vmaC* conditional strain
vmaC-*tet*-p4	CCGCTTGAGCAGACATCACCATGTCCAAGT CGAACAAGT	
tet-ptrA-up	TCTTGCATCTTTGTTTGTATTATACTGTC	*Tet-vmaC*

tet-ptrA-down	GGTGATGTCTGCTCAAGCGG	conditional strain confirmation
vmaC-NiiA-p3	GTAACGCCAGGGTTTTCCTGTGAATCAATTATGCAGT	*NiiA-vmaC* conditional strain
vmaC-NiiA-p4	GCGTTGAGACTTCGTCACGATGTCCATCGAACAAGT	
NiiA-fusion-up	TGCTCTGATGCCGCATAGGCTCAGAGTCTACAGCTG	*NiiA-vmaC* conditional strain confirmation
NiiA-down	CGTGACGAAGTCTCAACGC	
vmaC-alc-p3	GAGGCCTCTAGATGCATGCTCGAGCGTGAATCAATTATGCAGT	*alc-vmaC* conditional strain
vmaC-alc-p4	GCTTATCGATACCGTCGACCTCGAGATGTCCAAGTCGAACAAGT	
PyrG-*alc*-up	GCTCGAGCATGCATCTAGAGGCCTC	*alc-vmaC* conditional strain confirmation
PyrG-*alc*-down	CTCGAGGTCGACGGTATCGATAAGC	
GFP+PyrG-F	GGAGCTGGTGCAGGCGCTGG	Fusion PCR for GFP
GFP+PyrG-R	CTGTCTGAGAGGAGGCACTGATG	
vmaC-GFP-p1	ACGTCCGCTGAGCTTCTAT	*vmaC*-GFP strain construction
vmaC-GFP-p2	GTTTTGCTAGTCCTTCCTGC	
vmaC-GFP-p3	CCAGCGCCTGCACCAGCTCCATTGAATTCAAACTCGTAG	
vmaC-GFP-p4	CATCAGTGCCTCCTCTCAGACAGTGATGGCGGGATGATGCAC	
vmaC-GFP-p5	TCTTCCTCCGATGCTCCA	
vmaC-GFP-p6	GCACGCCTTTGACAGTTTTG	
RFP-up	GCCTCCTCCGAGGACGTCAT	Fusion PCR for RFP
RFP-down	TTAGGCGCCGGTGGAGTGGC	
CccA-RFP-p1	GCAAGAGGAGCTACACAATG	*CccA*-RFP strain construction
CccA-RFP-p2	GGATAATTGGAGACTGGACG	
CccA-RFP-p3	ATGACGTCCTCGGAGGAGGCAGCAGAACCATGGTCGATC	
CccA-RFP-p4	GCCACTCCACCGGCGCCTAATGATTTCATGATACCACAGC	
CccA-RFP-p5	GTGATATGGCGGTACGCT	
CccA-RFP-p6	GTTTCACCTCTTCTGCTGGA	
aeqS-F	ATGACCTCCAAGCAGTAC	Diagnosis PCR for

aeqS-R	TTAGGGGACGGCACCGCCGTA	aeqS
