# Supplementary figures and images for: Subunit C of V-ATPase-VmaC Is Required for Hyphal Growth and Conidiation in A. fumigatus by Affecting Vacuolar Calcium Homeostasis and Cell Wall Integration
Source: J Fungi (Basel). 2022 Nov 17;8(11):1219. doi: 10.3390/jof8111219 (PMC9699406; doi:10.3390/jof8111219)

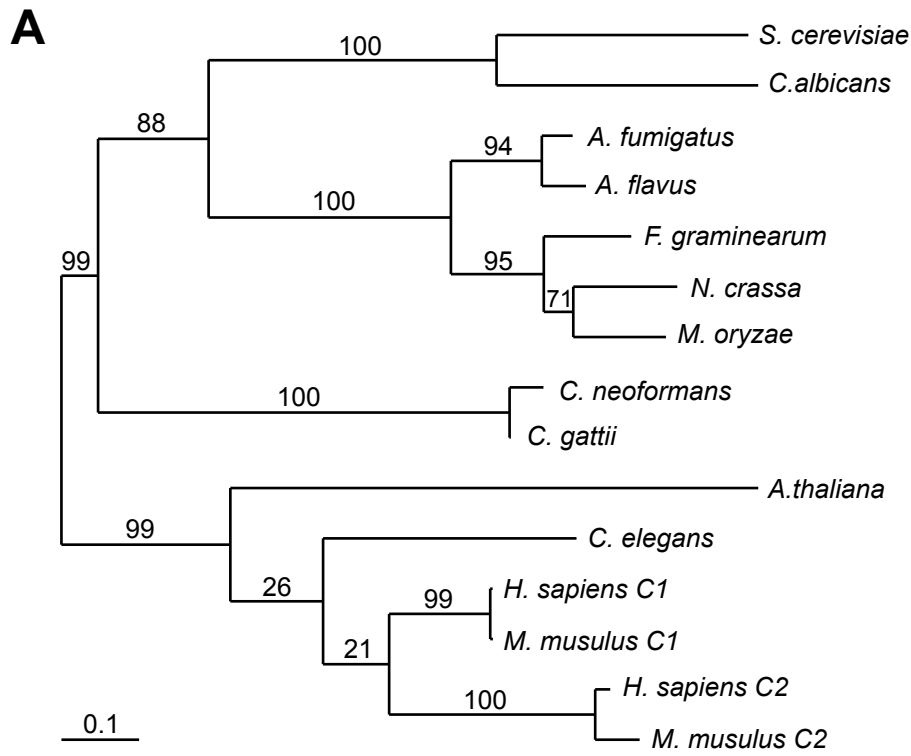

**B**

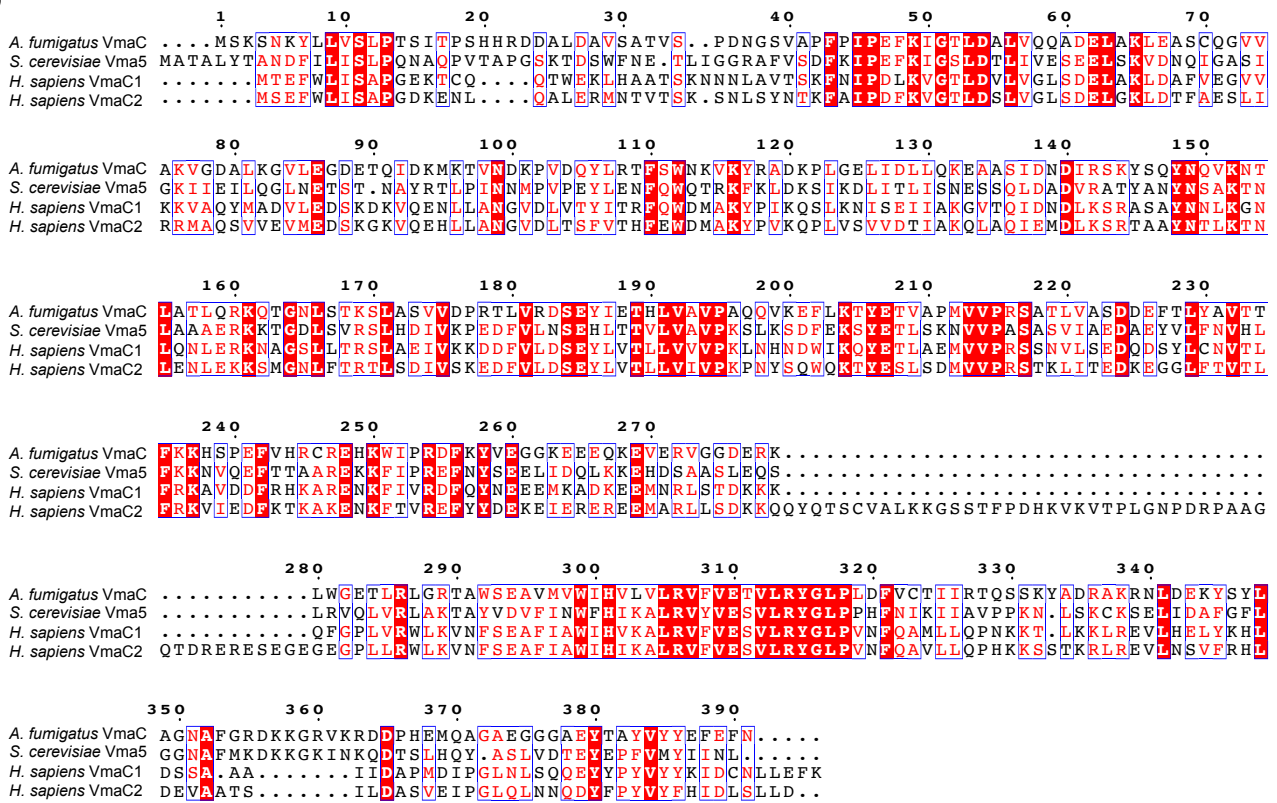

Supplement: Supplementary file 1 [file jof-08-01219-s001.zip › Fig S1.pdf]

A

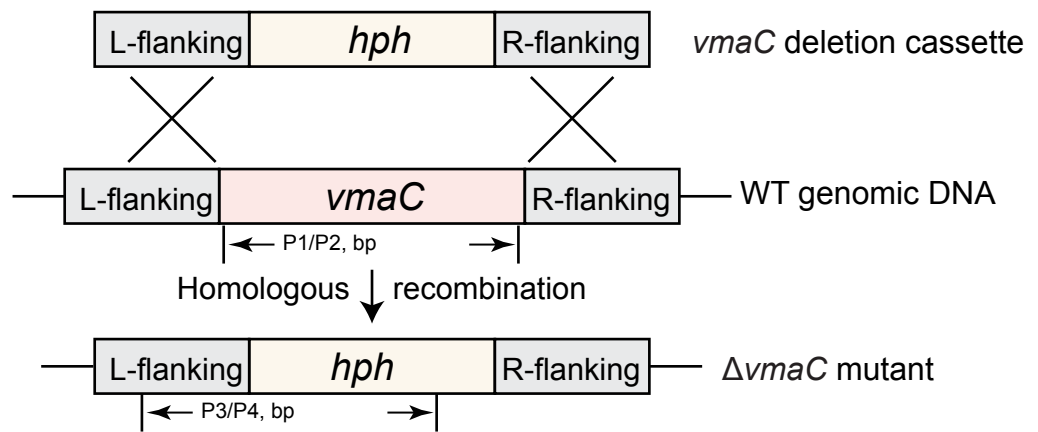

B

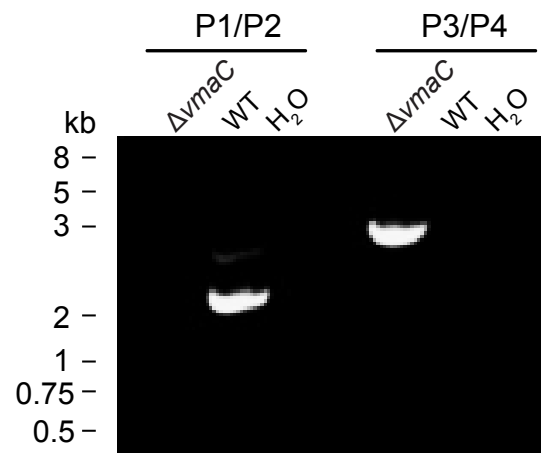

Supplement: Supplementary file 1 [file jof-08-01219-s001.zip › Fig S2.pdf]

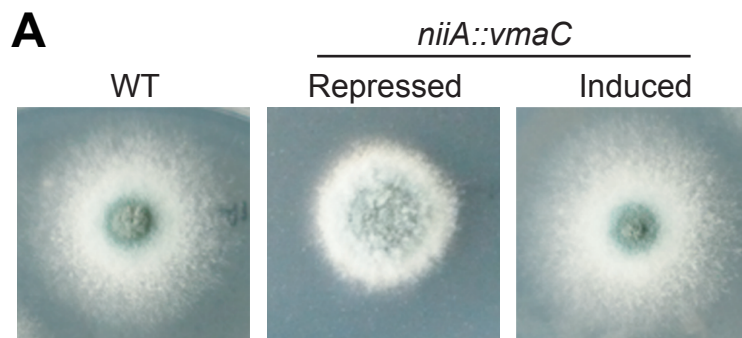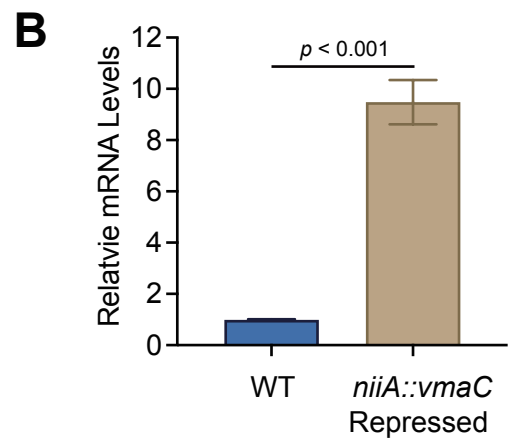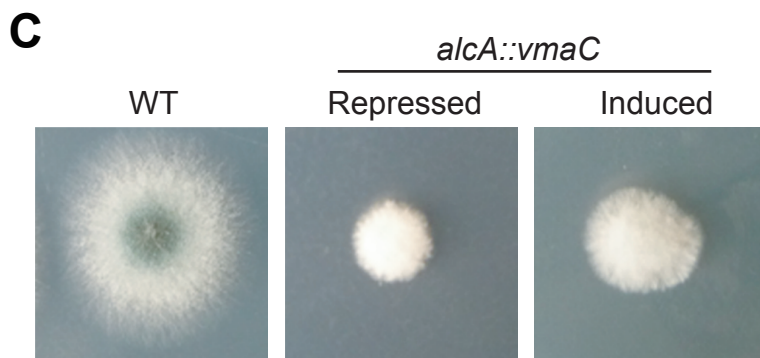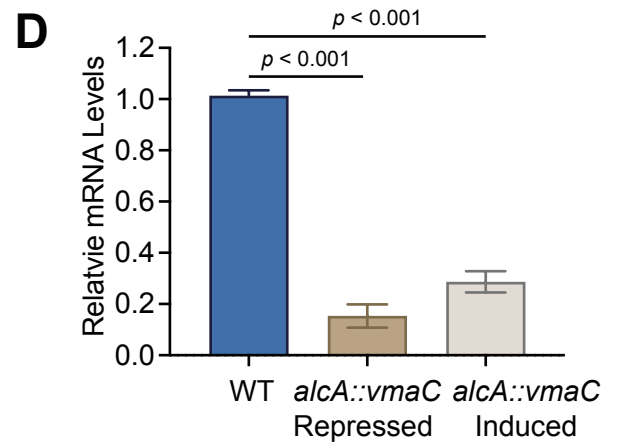

Supplement: Supplementary file 1 [file jof-08-01219-s001.zip › Fig S3.pdf]

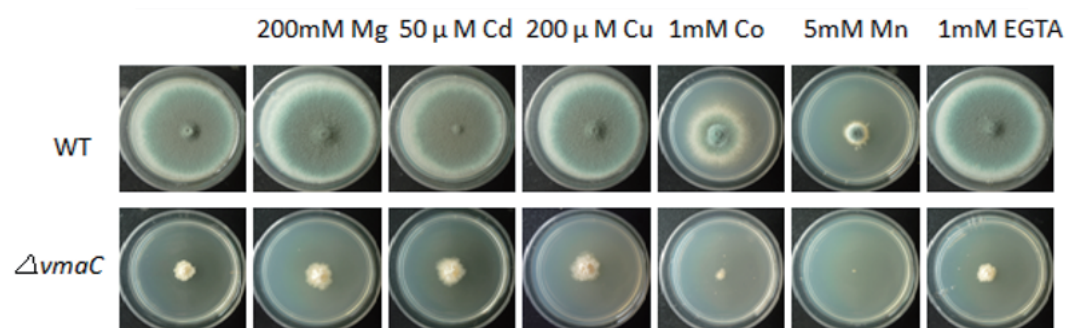

Supplement: Supplementary file 1 [file jof-08-01219-s001.zip › Fig S4.pdf]
